# Epidemiological Aspects and High Magnitude of Human Visceral Leishmaniasis in Ceará, Northeast of Brazil, 2007-2021

**DOI:** 10.1590/0037-8682-0684-2021

**Published:** 2022-05-20

**Authors:** Kellyn Kessiene de Sousa Cavalcante, Kelvia Maria Oliveira Borges, Francisco Roger Aguiar Cavalcante, Francisco Gustavo Silveira Correia, Caroline Mary Gurgel Dias Florêncio, Carlos Henrique Alencar

**Affiliations:** 1 Universidade Federal do Ceará, Faculdade de Medicina, Programa de Pós-Graduação em Saúde Pública, Fortaleza, CE, Brasil.; 2 Secretaria de Saúde do Estado do Ceará, Coordenadoria de Vigilância e Prevenção em Saúde, Fortaleza, CE, Brasil.; 3 Colégio Militar de Manaus, Sistema Colégio Militar do Brasil, Manaus, AM, Brasil.

**Keywords:** Visceral leishmaniasis, Morbidity and mortality indicators, Epidemiological surveillance, Epidemiological monitoring

## Abstract

**Background:**

Human visceral leishmaniasis HVL is endemic to 75 countries. The state of Ceará, the Northeast region of Brazil, is of great sanitary importance for the transmission of HVL, and it stands out as an area of interest for epidemiological aspects and control strategies. This study aimed to characterize HVL in relation to epidemiological aspects, composite incidence, and mortality rates in the state of Ceará, Brazil, from 2007 to 2021.

**Methods:**

This ecological study used temporal and spatial cuts of HVL data from the notifiable diseases information system. Epidemiological indicators such as incidence, mortality, and composite indices of incidence and mortality were calculated according to the Ministry of Health standardization.

**Results:**

There were 6,775 confirmed cases, with high incidence coefficients in 2009 6.96 cases/100,000 inhabitants and 2011 9.83 cases/100,000 inhabitants, and the highest mortality rate in 2011 6.96 deaths/100,000 inhabitants. The composite index of incidence and mortality identified municipalities in the Northern, Northwestern, and Southern regions of Ceará as having the highest risk of HVL.

**Conclusions:**

HVL remained endemic throughout the study period, with epidemiological indicators and risk of transmission expressing high magnitude, mainly in the Northeast, Northwest, and South regions of Ceará.

## INTRODUCTION

Human visceral leishmaniasis HVL is a chronic systemic anthropozoonosis caused by a protozoan of the genus *Leishmania* and is transmitted by *Lutzomyia longipalpis* ^1^. Endemic in 75 countries accounts for 90% of the reported cases in Brazil, India, South Sudan, Sudan, Ethiopia, Kenya, and Somalia^1^. 

The Brazilian Ministry of Health proposes that municipalities that transmit HVL develop and periodically analyze epidemiological and operational indicators to help plan and adopt timely measures for disease prevention and control^2^. 

Health indicators provide evidence on the health situation, identify vulnerable groups of people, stratify epidemiological risk, identify critical areas, and monitor the response capacity of actions and services^3^.

In areas with the transmission, periodic analysis of epidemiological and operational indicators should be carried out to evaluate the effectiveness of control measures and the progression of epidemiological situations, such as a change in incidence, mortality, case fatality, and changes in transmission areas that may worsen the problem^2^.

Currently, the Brazilian Ministry of Health uses surveillance and control measures based on at-risk areas. In addition, it incorporates silent municipalities into surveillance actions to avoid or minimize problems related to the disease in areas without transmission^2^. 

Control measures were different and appropriate for each location^4^. Actions are carried out in an integrated manner against the vector, reservoirs, and environment for early diagnosis, effective patient management, and integration between medical and veterinary services^5^. Given this, the Brazilian Ministry of Health has adopted the risk stratification of visceral leishmaniasis with aggregation of social and environmental variables and epidemiological indicators to support the definition of public policies, prioritizing surveillance, and control actions in recent years^4^.

Risk stratification is important for surveillance as it provides knowledge about health problems and supports managers and health professionals in adopting actions and targeting and prioritization of areas to be worked on^1^.

HVL is a weekly compulsory notification disease in Brazil, which is considered one of the four countries with the highest number of cases, with 14% of world cases and 97% of cases in America^1^. In 2019, 2,529 cases were registered in 920 municipalities in 22 of 27 federative units, with an incidence of 1.22 cases/100,000 inhabitants^1^. Furthermore, the Northeast region had the largest national casuistry 1,251; 49.46%, in which the states of Maranhão 390; 31.17%, Piauí 140; 11.19%, and Ceará 258; 20.62% stood out^6^.

The state of Ceará, endemic to HVL since the 1930s^6^, developed the Visceral Leishmaniasis Control Program covering human epidemiological surveillance, surveillance and control of the canine reservoir, chemical control of the vector, and health education^6^. Ceará is of great sanitary importance for the transmission of HVL^7^ and stands out as an area of interest for epidemiological aspects and the analysis of control strategies for this neglected anthropozoonosis.

HVL presents various clinical manifestations from asymptomatic, with a classic clinical picture of parasitosis, such as fever, pancytopenia, hypergammaglobulinemia, hepatosplenomegaly, and lymphadenopathy, to severe impairment of the general condition, which may lead to death if there is no timely treatment^1^ ^,^ ^8^. Therefore, case characterization is necessary to understand this disease and its use in surveillance and control program^9^.

We aimed to characterize HVL cases in terms of epidemiological aspects and composite indices of incidence and mortality in the state of Ceará, Brazil, from 2007 to 2021.

## METHODS

We conducted an ecological study with spatial analysis of temporal cuts of HVL cases and deaths between January 2007 and December 2021. An HVL case was considered an affected individual residing in the state of Ceará and diagnosed using rapid immunochromatographic, parasitological, or immunological tests by indirect immunofluorescence reaction IIFR. Notifications with a municipality of residence outside the state of Ceará and those with an inconsistent diagnostic field were excluded. 

**FIGURE 1:**
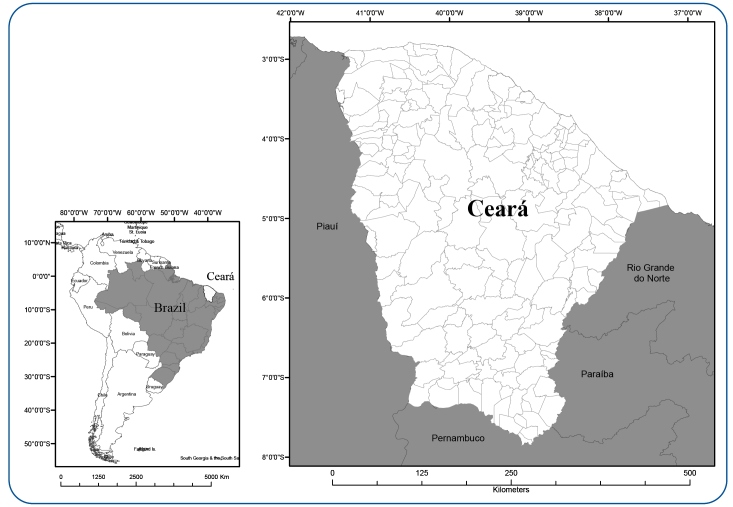
Map of Ceará State location in Brazil and Latin America.

The state of Ceará, located in the Northeast region of Brazil, is home to a population of approximately 9 million inhabitants 2021 estimate^10^ in 184 municipalities. Rainfall has an irregular temporal distribution and is more concentrated in the first 4 months. The maximum temperatures range from 29.4 °C March to 30.7 °C November, and the savanna is the predominant biome^10^ Figure 1.

The data source was the Brazilian Notification Information System of Diseases *Sistema de Informação de Agravos de Notificação - Sinan* in Portuguese. The selected sociodemographic variables were: age group <1, 1*-*4, 5*-*9, 10*-*19, 20*-*49, 50*-*79, and ≥80 years, sex male or female, level of education illiterate, complete elementary, middle level, and high level, race/color brown, white, black, indigenous, and yellow, zone of residence urban, rural, and peri-urban, clinical manifestations fever, weakness, splenomegaly, and weight loss, diagnosis rapid immunochromatographic test, parasitological, and immunological, type of treatment pentavalent antimonial and liposomal amphotericin B, and disease progression cure and death from visceral leishmaniasis. These variables were described using absolute and relative frequencies.

Additionally, epidemiological indicators of mortality and morbidity and composite indices of incidence and mortality of HVL were used. Epidemiological indicators were calculated according to the methods standardized by the Brazilian Ministry of Health^2^. Incidence coefficients were calculated by dividing the number of new HVL cases by the population of each municipality for each year of the study and multiplying the result by 100,000. Mortality coefficients were calculated by dividing the number of HVL deaths by the population for each year of the study and then multiplying the result by 100,000. All indicators were standardized by age group using a direct method. Population data were obtained from the Unified Health System Informatics Department *Departamento de Informática do Sistema Único de Saúde - Datasus* in Portuguese^11^.

Thematic maps were created to evaluate the measure of HVL risk in the various municipalities of Ceará. To reduce random errors and improve the stability of the data while making the maps, the indices were calculated in five blocks of triennium periods: 2007-2009; 2010-2012; 2013-2015; 2016-2018, and 2019-2021. The incidence indices provide information on the magnitude of the disease based on the average number of cases and incidence. On the other hand, mortality indices provide information on the severity of the disease in a given population based on the average number of deaths and mortality. This increases the risk of death due to the disease.

The calculation of the composite index follows some steps described as follows^12^.

1. For each municipality, the average number of cases in the 3-year period was calculated.
2. For each municipality, the average incidence coefficient in the 3-year period was calculated.
3. For each triennium, the mean and standard deviation of cases were calculated.
4. For each triennium, the mean and standard deviation of the incidence coefficients were calculated.
5. The normalized value of cases for each municipality was obtained by subtracting the mean number of cases for the triennium from the mean number of cases. This result was divided by the standard deviation of the number of cases in the triennium.
6. The normalized values of the incidence coefficients for each municipality were obtained using the same algorithm cited above, using the incidence coefficient for the 3-year period.
7. The normalized values of the cases and incidence coefficients for each municipality were then added. This value is known as the composite index.
8. Finally, the composite index values were sorted in ascending order and ranked into five natural breakpoints Jenks.

The classes of natural breaks were based on inherent groupings in the data. The spatial analysis program ArcMap identifies breakpoints by choosing the best group with similar values and maximizing the differences between classes, whose boundaries are defined when there are relatively large jumps in the data values^13^. Classification into predefined categories was used according to the Brazilian Ministry of Health: low, medium, high, intense, and very intense^1^. These categories can be compared, regardless of their numerical values; thus, the intensities of the respective indicators in different areas can be identified.

The same procedure was performed for the number of deaths and the mortality coefficients. Thus, one can obtain composite indices for areas with risk of transmission and death in the population with HVL in the state of Ceará. The values calculated for these indices are important for measuring and defining disease transmission intensity and direct control actions. 

The variables’ frequencies and the composite indices’ calculation were performed using the Stata software version 15.1 Stata Corp LP, College Station, TX, USA. Spatial autocorrelation of HVL incidence and mortality composite indices was measured using the Moran Local Index of Spatial Association LISA to identify the occurrence of clusters. We considered high-risk areas as a group of municipalities with high incidence and mortality. This analysis was performed using the TerraView 4.2. To create thematic maps, the ArcMap program version 9.2, with 184 municipalities of Ceará as the units of analysis for cartography.

The study was submitted to the Plataforma Brazil, with consideration and approval by the research ethics committee of the Federal University of Ceará on November 1, 2019 CAAE N^o^. 22785819.6.0000.5054. Without the identification of individuals, the study was based on publicly accessible secondary data.

## RESULTS

**FIGURE 2:**
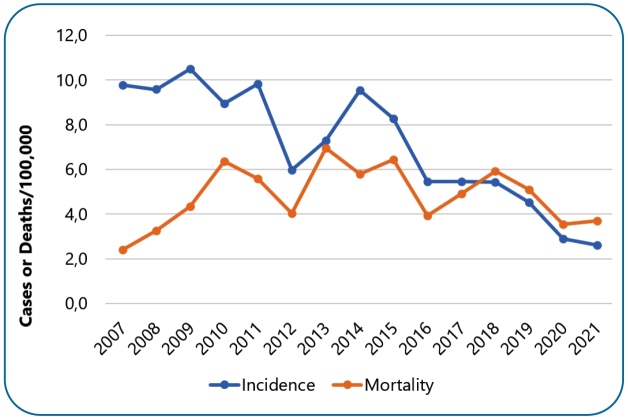
Temporal trends of human visceral leishmaniasis incidence and mortality rates/100,000 inhabitants, Ceará, 2007-2021. **Source:** SINAN data updated on 02/25/2022.

There were 6,755 new confirmed cases 89.15% using laboratory criteria, with an average of 450 cases per year and an incidence of 7.07 cases/100,000 inhabitants, and all 184 municipalities in Ceará registered at least one case of HVL. The standardized incidence coefficients showed a temporal trend of successive elevations and declines, with peaks in 2007 9.78, 2009 10.50, and 2011 9.83 cases/100,000 inhabitants. There were 394 deaths, with an average of 4.82 deaths/100,000 inhabitants. The standardized mortality rate also maintained a cyclical pattern with peaks in 2010, 2013, and 2015, with 6.37, 6.96, and 6.44 deaths/100,000 inhabitants, respectively Figure 2.

The highest frequency of cases was observed in the age group between 20 and 49 years 36.40%, followed by those aged between 1 and 4 years 21.29%. Additionally, the highest frequency of cases was in men 67.42%, mixed-race people 86.80%, those with incomplete primary education 9.59%, and those living in urban areas 72.33%. Clinical manifestations included fever 92.98%, weakness 73.09%, splenomegaly 71.83%, and weight loss 70.90%.

HVL positivity was diagnosed by immunochromatographic 52.89%, parasitological 34.34%, and immunological IIFR 17.45% tests. Treatment was prescribed for 6,619 97.99% patients, pentavalent antimonial 60.25%, and liposomal amphotericin B 15.83%.

Some municipalities located in the Northern General Sampaio and Morrinhos, Northwestern Sobral, Mucambo, Reriutaba, Ipueiras, and Ipaporanga, and Southern Barbalha and Nova Olinda regions stood out in the distribution of composite incidence and mortality rates. It is worth noting that Fortaleza, the capital of Ceará and the most populous city in the state, with approximately 2.7 million inhabitants, presented incidence indicators with transmission risks ranging from high to intense during the time intervals analyzed; and the composite mortality index classified as high in the period from 2007 to 2015 Figure 3.

**FIGURE 3:**
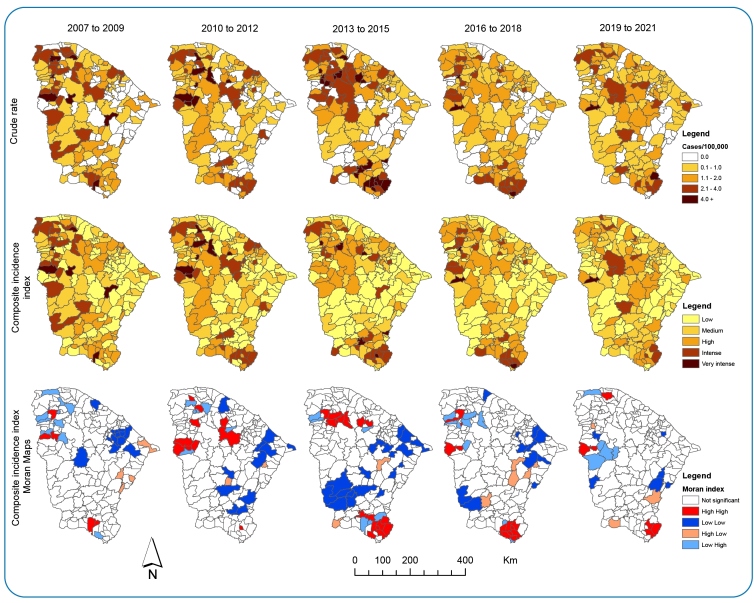
Maps of crude, composite, and Moran indices of incidence rates/100,000 inhabitants, Ceará, 2007-2021.

In the first period analyzed 2007 - 2019 it was possible to identify 34 18.48% municipalities with high incidence; of these, 10 had more than 1.23 cases per 100,000 inhabitants; and five had composite rates classified as very intense transmission, concentrated in three areas of the state: North; Northwest and South. During the second period 2010-2012, seven municipalities with a very intense risk of transmission were observed in the Northern and Northwestern areas of the state. The third period 2013-2015 showed very intense transmission in eight municipalities and very intense in 17 other municipalities 9.24%, with a cluster of high incidence rates in the Southern and Northwestern regions of the state. Low incidence rate clusters were identified in the Northeast and Southwest regions Figure 3.

There were more extensive clusters from 2013 to 2015, with significantly higher incidence values > 4.00 cases/100,000 population and a high risk of transmission high-high clusters, involving five municipalities in the Northwestern region of the state and eight municipalities in the Southern region Figure 3.

**FIGURE 4:**
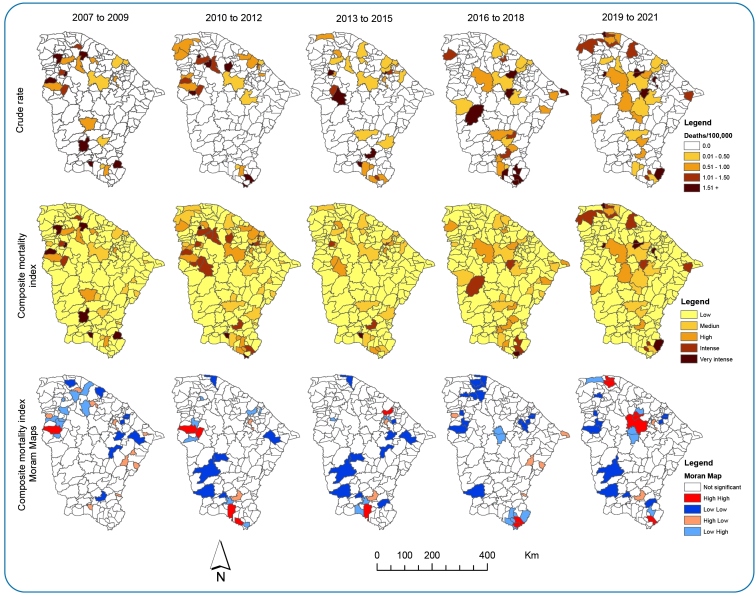
Maps of crude, composite, and Moran indices of mortality rates/100,000 inhabitants, Ceará, 2007-2021.

The composite mortality indices presented five municipalities, with mortality classified as very intense in the Northwestern and Southern areas of Ceará in the first, second, and third periods. In the fourth period 2016-2018, there was a high-high cluster of two municipalities was located in the South of the state. However, there was an isolated distribution of the composite mortality index in the Northern, Northwestern, and Southern regions of the state Figure 4.

## DISCUSSION

Ceará presented frequent and high occurrences of HVL cases between 2007 and 2021, with endemic transmissions in municipalities located mainly in the Northeast, Northwest, and South regions. Allied with the composite indices of incidence and mortality, these three regions present municipalities with high urbanization rates, an intense flow of people, and potential reservoirs. In addition, these municipalities are located within the expansion of their metropolitan regions. In these areas, anthropic action on the environment and the adaptation of the vector to the urbanized space led to a large proportion of people being at risk of infection with HVL^7^ ^,^ ^14^.

The World Health Organization WHO considers HVL one of the five priority neglected diseases for elimination worldwide^9^. Considering the complexity of disease transmission, epidemiological surveillance is one of the priorities of the national HVL control program to reduce incidence and case-fatality rates. Timely diagnosis and treatment, management of the reservoir population, and etiological agents are the most impactful actions to reduce the risk of transmission^4^.

Ceará had 147 municipalities with HVL transmission, classifying it as an area with a high risk of transmission. Of these municipalities, 33 are considered priorities for the direction of surveillance and control actions, with 2 classified as very intense transmission Fortaleza and Ipaporanga, 4 as high transmission Barbalha, Crato, Granja, and Assaré, and 27 as medium transmissions^6^. In addition, high-risk municipalities may have structural deficiencies, such as high turnover of health professionals, difficulty controlling the transmission of leishmaniasis in canine reservoirs, and lack of health education.

Fortaleza presented a transmission risk that varied from high to intense in the incidence indicators of the five analyzed trienniums. Furthermore, the number of seropositive dogs and phlebotomine sandflies is concerning, considering that canine infection precedes the occurrence of human cases^16^. Therefore, the main determinants of the HVL epidemic levels in Fortaleza are the close coexistence of the human/dog binomial and the increase in the vector's population density^17^.

The behavior of successive increases and decreases in the incidence and mortality coefficients was also identified in the city of Aracaju from 1999 to 2008 and in the state of Piauí, located in Northeastern Brazil, from 2007 to 2011^18^ ^,^ ^19^. In addition, a study on the HVL incidence coefficient trends in the city of Araçatuba São Paulo between 1999 and 2015 found that the reduction in cases probably occurred through the implementation of control actions, including better organization of health services for diagnosis and early treatment of cases, reduction of the sand fly population, elimination of reservoirs, and health education activities^20^.

In Ceará, other control actions implemented included the development of a state action plan for the intensification of visceral leishmaniasis surveillance and control actions until 2022, with proposals for health education and strengthening of epidemiological surveillance, guidance to professionals and the population through the dissemination of technical reports, and technical support to municipalities for monitoring the proportion of HVL cases confirmed using laboratory criteria, a strategy of the state panel of indicators^6^.

There were more notifications of the disease among young adults and men living in urban areas. The high susceptibility of children may be associated with humoral and cellular immaturity^21^. The data regarding the sex of affected individuals corroborate those presented in the states of Alagoas^22^ and Pará^23^. The predominance of the disease in men is justified by the presence of sociocultural sex differences in healthcare-seeking behaviors, in which men usually do not seek health treatment at the beginning of the diseases^24^.

Some studies have shown that HVL mainly affects people of low socioeconomic status once these conditions cause high exposure to the vector, which enables the transmission of anthropozoonosis^8^ ^,^ ^25^. Here, the most significant involvement of mixed-race people and low educational levels occurred in the border and peripheral municipalities. There was a high incidence of cases in populous municipalities with unfavorable socioeconomic conditions. These factors determine the proliferation and maintenance of the vector *Lutzomyia longipalpis*, which has adapted to the peridomestic areas of urban areas with precarious sanitary conditions^16^ ^,^ ^21^. 

The prevalent cases in urban areas found in this study reflect the historical reality of the disease, in which the urban population in Brazil has reached 85%, which has led to the emergence and reemergence of anthropozoonoses such as HVL^21^. Ceará is the third most populous state in the Northeast region and eighth in Brazil, with 75% of residents in urban areas^11^. Furthermore, there is accentuated deforestation and a constant migratory process in the urban areas of cities^17^. Large wild areas with economic exploitation, proximity to dwellings, high population density, and high susceptibility to infection have contributed to the rapid expansion of HVL in the periphery of urban centers^23^.

Approximately 90% of the cases were confirmed using laboratory criteria. This indicator relates to the good operational capacity of the laboratory service, which improves the specificity of the surveillance system^4^. Diagnosis is made by the Central Public Health Laboratory of the state of Ceará Lacen/SESA-CE, but rapid immunochromatographic tests can be used in health units in priority municipalities^6^. 

In agreement with other studies carried out in Aracaju-SE 1999-2008^18^, Bauru-SP 2004-2012^21^, and Alagoas 2007-2013^22^, most affected patients exhibited classic clinical manifestations, including fever, hepatosplenomegaly, weakness, and weight loss. Therefore, the first choice of drug in Ceará is pentavalent antimonial. However, research on the efficacy of drugs currently used for the treatment of this disease is scarce^16^. Therefore, routine diagnosis, treatment, and monitoring of patients need to be implemented in all areas with the transmission or at risk of transmissions^26^ ^,^ ^27^.

Ceará has environmental characteristics that attract various enterprises, especially the activities of wind energy companies, commerce, agriculture, and an increase in tourism, which favors a great dispersion of the disease^16^. However, this can lead to outbreaks of various infectious diseases resulting from the expansion of the urban frontier. Furthermore, this expansion into wild environments is conducive to the presence of a vector that often occurs in a disorderly manner, which directly influences the increase in cases of HVL^3^.

Inadequate social conditions may favor the spread of Leishmania^14^. Local social inequality increases the contact between humans and domestic animals, which contributes to an increase in the number of pet-related diseases and the incidence of zoonosis^15^. From 2004 to 2016, a study described the epidemiology of leishmaniasis before and after the peace agreement in Colombia. The annual incidence of leishmaniasis registered a downfall, with an annual percentage change of 17.7% after the peace treaty^15^. 

The increase in deaths after 2012 may be due to a possible delay in diagnosis and treatment, identified as risk factors for death^18^. Among the municipalities that showed high case fatality rates, Sobral stood out in Ceará, and 6.21% of the patients affected with HVL died. On the contrary, in Fortaleza, a low case fatality rate 5.84% was found, similar to that detected in this study^18^. 

A study conducted between 2011 and 2015 showed that Sobral, one of the municipalities with high indicators in this study, was classified with an intense transmission rate, being the second municipality with the highest number of notified cases in Ceará, after Fortaleza^27^. Sobral and Fortaleza are municipalities with high population flow, a growing urbanization process, rural emptying, and periodic droughts, which may have contributed to the high incidence and mortality rates detected.

The magnitude of morbidity and mortality is characterized by a sudden and rapid increase in the number of cases. These increases were primarily associated with social and environmental factors^9^. However, the increase in mortality after 2013 may be due to a possible delay in diagnosis and treatment, identified as risk factors for death^18^. HVL is lethal in approximately 95% of patients if left untreated^28^. 

It is worth noting that the municipalities of the state of Ceará with the highest incidence also had the highest mortality coefficients, and some of them Ipaporanga, Barbalha, and Sobral are already classified as a priority in terms of transmission risk; however, they are targets for special attention in terms of great surveillance, timely diagnosis, and adequate treatment^6^. 

On the other hand, other municipalities are not in this new stratification of the Ministry of Health in Brazil^4^, pointing to a necessary intensification of surveillance and control actions for VHL in the state. More effective control of HVL in Ceará is necessary, aiming at the early identification of cases and minimizing transmission to reduce morbidity and mortality in the identified risk areas.

This research had some limitations related to using secondary data from the SINAN, such as some fields having inadequate filling out and missing or incomplete information. However, given a large number of notifications in Ceará, these shortcomings did not compromise the data.

HVL remained endemic throughout the study period, with epidemiological indicators and transmission risk expressing a high magnitude in Ceará. The highest incidence and mortality rates were found, especially in the most populated municipalities, indicating urbanization of the disease. The data indicate the need for continuity and intensification of HVL surveillance and control actions, especially identifying priority areas to assist local management and seeking to break the chain of transmission of the disease.
